# Digital literacy level and associated factors among health professionals in a referral and teaching hospital: An implication for future digital health systems implementation

**DOI:** 10.3389/fpubh.2023.1130894

**Published:** 2023-04-11

**Authors:** Masresha Derese Tegegne, Binyam Tilahun, Adane Mamuye, Hailemariam Kerie, Fedlu Nurhussien, Endalkachew Zemen, Aragaw Mebratu, Girma Sisay, Redet Getachew, Henok Gebeyehu, Abiy Seyoum, Selamsew Tesfaye, Tesfahun Melese Yilma

**Affiliations:** ^1^Department of Health Informatics, Institute of Public Health, College of Medicine and Health Sciences, University of Gondar, Gondar, Ethiopia; ^2^eHealth Lab Ethiopia, University of Gondar, Gondar, Ethiopia; ^3^Department of Computer Science, College of Informatics, University of Gondar, Gondar, Ethiopia; ^4^ICT Directorate, University of Gondar, Gondar, Ethiopia; ^5^Department of Information System, College of Informatics, University of Gondar, Gondar, Ethiopia

**Keywords:** digital literacy, health professionals, associated factors, Northwest Ethiopia, referral and teaching hospital, digital health systems

## Abstract

**Background:**

In Ethiopia and other developing countries, electronic medical record systems and other health information technology are being introduced. However, a small proportion of low-income countries have successfully implemented national health information systems. One cause for this can be the lack of digital literacy among medical practitioners. As a result, this study aimed to assess health professionals’ digital literacy level and associated factors in Northwest Ethiopia.

**Method:**

A quantitative cross-sectional study was employed among 423 health professionals working in a teaching and referral hospital in Northwest Ethiopia. We modified and applied the European commission’s framework for digital competency to assess the level of digital literacy among health professionals. We used stratified random sampling with proportional allocation to the size of the departments in the hospital to select study participants. Data were collected using a semi-structured, self-administered, and pretested questionnaire. Descriptive and binary logistic regression analysis techniques were used to describe respondents’ digital literacy level and identify its associated factor, respectively. The odds ratio with 95% CI and value of p were used to assess the strength of the association and statistical significance, respectively.

**Results:**

Out of 411 participants, 51.8% (95% CI, 46.9–56.6%) of health professionals had adequate digital literacy. Holding a master’s degree (Adjusted OR = 2.13, 95% CI: 1.18–3.85), access to digital technology (AOR = 1.89, 95% CI: 1.12–3.17), having training in digital technology (AOR = 1.65, 95% CI: 1.05–2.59), and having a positive attitude towards digital health technology (AOR = 1.64, 95% CI: 1.02–2.68) were found to be significant factors associated with health professionals digital literacy level of health professionals.

**Conclusion:**

Low level of digital literacy among health professionals was observed, with nearly half (48.2%) of them having poor digital literacy levels. Access to digital technology, training on digital technology, and attitude toward digital health technology were significant factors associated with digital literacy. It is suggested to increase computer accessibility, provide a training program on digital health technology, and promote a positive attitude toward this technology to improve the deployment of health information systems.

## Background

Digital technology has a tremendous effect on improving the quality of health services in both developed and developing nations by enhancing the accessibility of health information and creating an efficient service provision (1). In high-income countries, digital health solutions are gradually being implemented in healthcare settings (2). However, only 35% of lower-middle-income nations and 15% of low-income countries have implemented national electronic health record systems in hospitals (3, 4). Most health facilities rely on paper-based systems, leading to inaccuracies in data management practice, which impacts the quality of healthcare delivery (5, 6).

Evidence suggests that healthcare information systems can enhance the quality of healthcare delivery and are expected to be implemented in all healthcare services (7). Therefore, using and operating information technology is a requirement for healthcare workers. Digital health technology refers to the collection, sharing, and analysis of health information using digital information, data, and communication technologies to enhance patient health and healthcare provision (8, 9). Examples include computers, tablets, smartphones, digital medical equipment, smartwatches, and other digital technology. The term digital literacy refers to “the ability to use technology to participate in and contribute to modern social, cultural, political, and economic life” (10). For the successful transformation of healthcare delivery, digital literacy is a prerequisite (11). Technologically savvy health workers can better manage their patients (12). Excellent digital literacy can lead to increased readiness for electronic health record systems (13). In turn, this might improve healthcare systems’ efficiency and long-term viability.

Ethiopia has several eHealth project initiatives underway, and most of the nation’s previously implemented health information systems faced sustainability challenges (14, 15). The main reason for low adoption or sustainability issues for EHR systems is a lack of pre-implementation efforts, such as a lack of digital literacy among health professionals (16–18). As a result, identifying areas and requirements before implementation could assist in determining the areas of focus that must be addressed throughout implementation.

Various studies are being conducted worldwide to evaluate healthcare professionals’ knowledge, perception, and willingness in using digital health tools (19, 20). Evidence also supports medical professionals’ adoption of digital health technologies for clinical services in response to the COVID 19 pandemic (21, 22). Previous studies in similar situations demonstrate that a lack of digital literacy among health practitioners is a significant factor in digital health system failure (5, 18, 23). Healthcare practitioners in low-income nations such as Ethiopia should have at least a basic knowledge of digital technology to implement e-health systems successfully (16). According to studies, healthcare workers’ digital skill gaps must be bridged for technology to be transferred to the point where health service quality can be maintained (5, 24).

Currently, the University of Gondar, in collaboration with the Ethiopian Ministry of Health (FMOH) and Digital Health Activities (DHA) agreed to customize and implement an Electronic Medical Record (EMR) at the University of Gondar Specialized and Comprehensive Hospital. The hospital serves as the sole referral center in Northwest Ethiopia, with a range of speciality healthcare services and a teaching and research center. Despite the range of services that it provides, its information system is not yet computerized. As a result of the adoption of e-health initiatives, the University of Gondar specialized hospital has been chosen as the pilot study for the EMR deployment. As far as we know, there are limited evidences available regarding the level of digital competency among healthcare professionals working in hospital settings. Hence, before starting a costly implementation, it is essential to assess the level of digital literacy of health professionals working at the implementation site. Therefore, this study aimed to evaluate the level of digital literacy and identify its associated factors among health workers at the University of Gondar Specialized and Comprehensive Hospital. Understanding the digital literacy of health workers could help take appropriate measures to successfully implement an EMR system at the hospital.

## Methods

### Study design and setting

An institutional-based cross-sectional study was conducted among health professionals at the University of Gondar specialized and comprehensive hospital from June 2 to June 25, 2022. The University of Gondar Specialized Hospital is located in the historic town of Gondar, northwest Ethiopia. Gondar is approximately 168 kilometres from Bahir Dar and 772 kilometres from Addis Abeba, the country’s capital. Nearly seven million people are served by the University of Gondar Comprehensive Specialized Hospital, one of the largest referral and teaching hospitals in the Amhara region (25). It employed 1,520 healthcare professionals across more than 20 departments to treat an average of 1,000 patients daily.

### Study sample and eligibility criteria

All health professionals working at the University of Gondar Specialized and Comprehensive Hospital were included in this study. However, health professionals with less than six months of experience, those who will not be present in hospitals for various reasons, and those on yearly leave during the data collection period were excluded from the study.

### Sample size determination and sampling procedure

The sample size was calculated using the single population proportion formula n=$\frac{{\left(Z\propto /2\right)}^{2}P\left(1-P\right)}{d^{2}}$ using the following assumptions (26). Since there is a study in Ethiopia addressing the digital literacy of health professionals working in primary health centers (*p* = 50%) (5), based on the assumptions of a 95% CI (z α/2 = 1.96), a 5% degree of precision (d), the final sample size becomes 384. A total of 423 healthcare professionals were included in the study after accounting for the 10% non-respondent rate. Each department served as a stratum in our department-based stratified random sampling design. A list of health professionals was used as the sampling frame. The level of digital literacy and related parameters were evaluated by proportionally allocating the sample to each department based on the number of healthcare providers. Participants in each department were then selected using a computer-generated simple random sampling technique.

### Study variables

The dependent variable in this study was the digital literacy level of the healthcare practitioners, while the independent variables were socio-demographic characteristics (sex, Age, Profession, Educational level, Work experience), technological variables (Access to digital technology, Internet access, Training on digital technology), organizational variables (Staff motivation and workloads), and attitudes towards the use of digital health technology.

### Measuring instruments

**Digital literacy:** Twenty-one item questions were adapted from the European Commission’s digital competency framework and used to assess health workers’ digital literacy (27). The digital literacy level is measured using a 5-point scale (Very Good, Good, Acceptable, Poor, and Very Poor) and separated into five primary components (Information processing, content creation, Communication, Safety, and Problem-solving). Since the data is not normally distributed, a median of the 21 questions about health professionals’ digital literacy level was calculated, and those who scored higher than the median were classified as having adequate digital literacy. In contrast, those who scored lower were classified as having inadequate digital literacy (5).

**Attitude towards digital health technology:** sixteen questions adapted from The Digital Health Scale were used to measure the health professionals’ attitude toward using digital health technology (28). The attitude toward digital health technology was measured using a 5-point scale (Strongly Agree, Agree, Neutral, Disagree, and Strongly Disagree). The item scores for each composite variable were added and divided by the number of items to create a composite variable ranging from scores 1 to 5 for the data analysis (29–31). As a result, the above three of final scores (strongly Agree, and Agree) were labelled as “Favorable attitude’. In contrast, final scores of three or less (strongly disagree, disagree, and neutral) were categorized as “Unfavorable attitude’ (31, 32).

### Data collection tool and quality control

The questionnaires used in this study were developed after a review of related literature (5, 27, 28). A self-administered and pretested questionnaire was developed in English to collect the required data. The questionnaires assess the level of digital literacy among health professionals and their attitudes toward digital health technology, socio-demographic variables, technological attributes, and organizational characteristics. The data collection process involved two supervisors and twelve data collectors. Supervisors and data collectors received three days of training to minimize ambiguity. A pretest was conducted outside the study area, in Gondar town health centers, with 10% of the study population. The pretest results were used to evaluate the data collection instrument’s validity and reliability. Cronbach’s alpha scores were used to assess the data collection instrument’s internal consistency. As a result, the Cronbach alphas for digital literacy level (0.97) and attitude toward digital technology use (0.91) were found to be within the acceptable range.

### Data processing and analysis

The Epi Data version 4.6 software packages were used for data entry, and the Statistical Package for Social Sciences (SPSS) version 25 was used for analysis. Descriptive statistics were computed to describe socio-demographic variables and health professionals’ digital literacy levels. Bivariable and multivariable binary logistic regression analyses were used to identify the relationship between the dependent and independent variables. Variables with a value of p less than 0.2 in the bivariable regression analysis were considered potential candidates for the multivariable regression analysis to assess their adjusted impacts on the dependent variables. An odds ratio with a 95 percent confidence level and *p*-value was calculated to determine the association’s strength and statistical significance. The cut-off value for all significantly associated variables was *p* < 0.05.

### Ethics approval and consent to participate

Ethical clearance and approval letters were obtained from the Institutional Review Board (IRB) of the University of Gondar with reference number (**Rfe. VP/RTT/05/571/2022**). After explaining the study’s objective, each health professional signed a written consent form. The University of Gondar’s specialized hospital also obtained a letter of support. Confidentiality and privacy were ensured during data collection by keeping participants anonymous.

## Results

### Socio-demographic characteristics

This study enrolled 411 health professionals, and the response rate was 97.1%. The participants’ average age was 30.3 years, with a standard deviation of +4.8 years, and a minimum and maximum age of 21 and 60, respectively. Of all participants, 240 (58.1%) were men, and 161 (39.2%) of the respondents identified as nurses. The majority of 336 (81.8%) health professionals had a degree or below in education, and 222 (54.0%) were married at the time. Regarding employment history, participants had an average of 6 years of experience (Table 1).

**Table 1:** Socio-demographic characteristics of respondents.

Socio-demographic variables	Category	Frequency	Percentage
Sex	Male	240	58.4%
	Female	171	41.6%
Age	30.3 ± 4.89		
Profession	Physician	89	21.7%
	Nurse	161	39.2%
	Pharmacy	18	4.4%
	Midwifery	51	12.4%
	Laboratory	32	7.8%
	Psychiatry	7	1.7%
	Public health	21	5.1%
	Physiotherapy	7	1.7%
	Optometry	9	2.2%
	Anesthesia	8	1.9%
	Radiology Assistant	8	1.9%
Marital status	Single	183	44.5%
	Married	222	54.0%
	Divorced	4	1.0%
	Widowed	2	0.5%
Educational level	Degree and Below	337	82.0%
	MSc and Above	74	18.0%
Work experience	6.78 ± 6.5		

### Technological and organizational factors

Most health professionals, 298 (72.5%), have access to digital devices like computers, tablets, smartphones, digital medical devices, smart watches, etc. Of all respondents, around 350 (85.1%) have access to a smartphone. A desktop computer, a laptop computer, and smartwatches are accessible to 154 (37%), 187 (45%), and 42 (10.2%) health professionals, respectively. However, only 51 (12.4%) health workers had access to one of the digital medical devices, such as an electronic (digital) vision chart, wearables, an auto-refractor, a scan biometer, glucose monitors and heart-rate monitors. Accessible digital medical equipment included glucose monitors, heart-rate monitors, wearables, an auto-refractor, a scan biometer, and an electronic (digital) vision chart. Nearly half, 221 (53.8%) of the respondents had access to one of the aforementioned digital technologies at work, and 332 (80.8%) of them had access to an internet connection (Table 2).

**Table 2:** Technological and ororganizational-relatedariables.

Variables	Category	Frequency	Percentage
Access to digital technology	No	113	27.5%
	Yes	298	72.5%
Which digital devices do you have access to?*	Desktop computer	154	37.5%
	Laptop computer	187	45.5%
	Smartphones	350	85.1%
	Digital medical devices**	51	12.4%
	Smartwatch	42	10.2%
Accessible digital technology in the workplace	No	190	46.2%
	Yes	221	53.8%
Internet access	No	79	19.2%
	Yes	332	80.8%
Where do you get access to the Internet?*	Private Wi-Fi and Mobile data	305	74.2%
	Internet cafe	88	21.4%
	Workplace	232	56.4%
Training in digital technology	No	196	47.7%
	Yes	215	52.3%
Motivation	No	180	43.8%
	Yes	231	56.2%
Number of Patients served per day	19.60 + 10.4		

*Multiple options possible, ** Glucose monitors, heart-rate monitors, wearables, an auto-refractor, a scan biometer, and an electronic (digital) vision chart.

Regarding organizational characteristics, health professionals claimed that they visit an average of 19 and more patients daily. From the total of 231 (56.2%) respondents, the staff is also driven to use digital technology for patient care. Furthermore, 215 (52.3%) health professionals have received training in digital technology.

### Attitude towards digital health technology

Of the total respondents in this study, 307 (74.7%) with (95% CI:70.4–78.9%) had a favourable attitude towards using digital health technology to provide patient care.

Table 3 shows that 194 (47.2%) health professionals agreed that booking an appointment on a computer or smartphone was more practical. A total of 185 (45.0%) respondents said using technology had improved healthcare. Around 44% of health professionals responded that they understand how to use digital health technology. Surprisingly, 157 (38.2) respondents asserted that video and telephone appointments with patients are just as effective as in-person meetings. Nearly half of the responders (209) (50.9%) believed that health technology benefits everyone. Similarly, 207 (50.4%) of participants expressed confidence in the confidentiality of medical records due to technology. Additionally, 170 (41.4) respondents were enthusiastic about the increased usage of technology in healthcare.

**Table 3:** Attitude towards digital health technology.

Attitude variables	SD (%)	D (%)	N (%)	A (%)	SA (%)
Making an appointment on a computer or smartphone would be more convenient for me.	27 (6.6)	59 (14.4)	46 (11.2)	194 (47.2)	85 (20.7)
I think using technology has improved healthcare	34 (8.3)	41 (10.0)	31 (7.5)	185 (45.0)	120 (29.2)
I really understand how to use health technology	22 (5.4)	74 (18.0)	80 (19.5)	178 (43.3)	57 (13.9)
Video and telephone appointments with my patients are as good as meeting them in person	38 (9.2)	84 (20.4)	80 (19.5)	157 (38.2)	52 (12.7)
Health technologies are easy to use	35 (8.5)	87 (21.2)	77 (18.7)	166 (40.4)	46 (11.2)
Patients and hospitals rely too much on technology	76 (18.5)	99 (24.1)	93 (22.6)	116 (28.2)	27 (6.6)
Technology could never replace real health professionals	44 (10.7)	91 (22.1)	83 (20.2)	131 (31.9)	62 (15.1)
I would like to see more use of technology in healthcare	29 (7.1)	61 (14.8)	62 (15.1)	170 (41.4)	89 (21.7)
Health technology is less likely to break down, and my work will not be affected	36 (8.8)	95 (23.1)	86 (20.9)	151 (36.7)	43 (10.5)
Health technology reduces human error	46 (11.2)	51 (12.4)	69 (16.8)	185 (45.0)	60 (14.6)
The thought of using an online appointment system makes me relaxed	48 (11.7)	50 (12.2)	72 (17.5)	177 (43.1)	64 (15.6)
The thought of new developments in health technology is exciting	16 (3.9)	69 (16.8)	71 (17.3)	196 (47.7)	59 (14.4)
I often use health technology	27 (6.6)	73 (17.8)	73 (17.8)	176 (42.8)	62 (15.1)
Health technology is good for everyone	17 (4.1)	42 (10.2)	58 (14.1)	209 (50.9)	85 (20.7)
I’m confident that technology will keep the medical records private	18 (4.4)	49 (11.9)	58 (14.1)	207 (50.4)	79 (19.2)
I enjoy using health technology	14 (3.4)	24 (5.8)	62 (15.1)	221 (53.8)	90 (21.9)

SD, Strongly Disagree; D, Disagree; N, Neutral; A, Agree; SA, Strongly Agree.

### Health professionals’ digital literacy level

**Figure 1:**
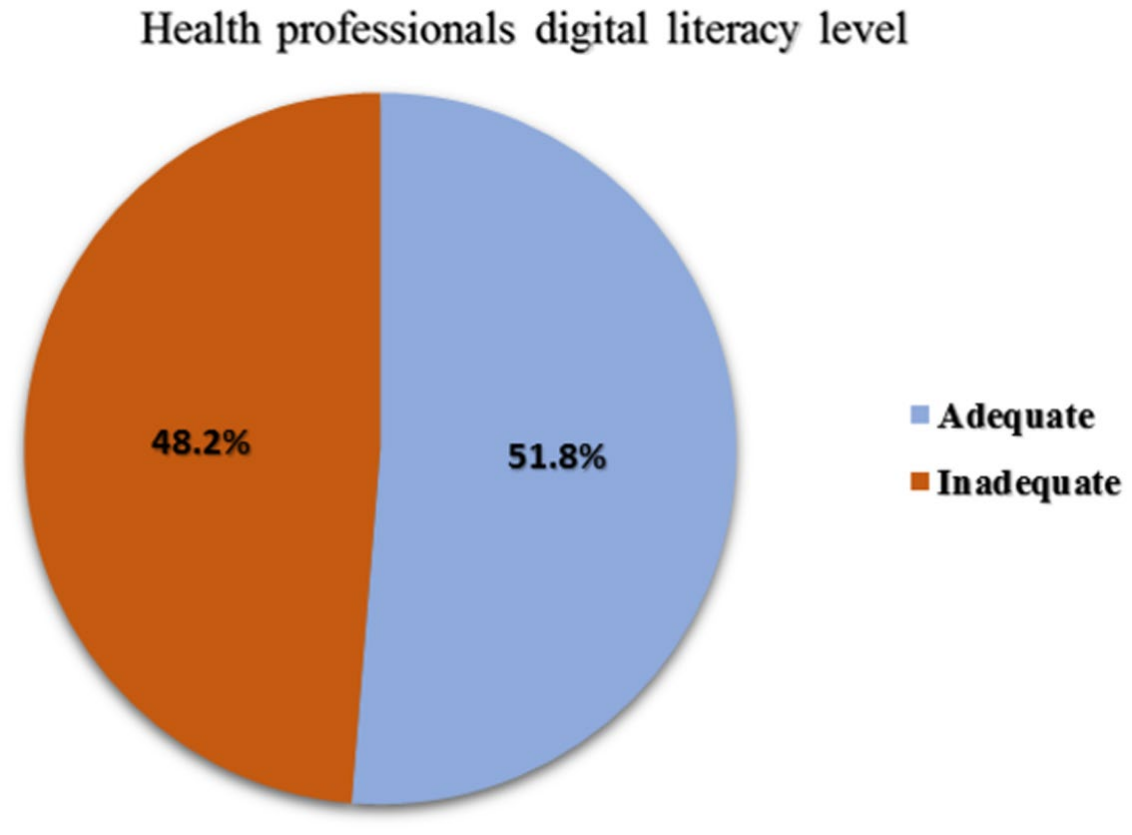
Health professionals’ digital literacy level.

Overall, 213 (51.8%) (95% CI, 46.9–56.6%) health professionals have adequate digital literacy levels (Figure 1). As shown in Table 4, the combination of five digital literacy components, information and data literacy, collaboration and communication literacy, literacy in creating digital content, safety, and problem-solving, was used to assess the respondents’ overall levels of digital literacy. Of the total, 233 (56.7%) had adequate information and data literacy. Regarding collaboration and communication, more than half of the health professionals, 223 (54.3%), possessed adequate literacy. Of the total respondents, 220 (53.5%) had adequate literacy in digital content creation. Additionally, 206 (50.1%) and 249 (60.6%) exhibited adequate literacy levels in safety and problem-solving, respectively.

**Table 4:** Components of health professionals’ digital literacy.

Digital literacy subscales	Median (±SD)	Poor	Good
Information and data literacy	10.00 ± 3.45	178 (43.3%)	233 (56.7%)
Communication and Collaboration	20.00 ± 6.48	188 (45.7%)	223 (54.3%)
Digital content creation	12.00 ± 4.33	191 (46.5%)	220 (53.5%)
Safety	14.00 ± 4.64	205 (49.9%)	206 (50.1%)
Problem-solving	12.00 ± 4.54	162 (39.4%)	249 (60.6%)

### Factors associated with health professionals’ digital literacy level

The factors affecting the level of digital literacy among health professionals were examined using bivariable and multivariable logistic regression analysis. In the bivariate analysis, sex, age, educational status, monthly income, work experience, access to digital technology, internet access, training on digital technology, organizational motivation, attitude towards digital technology, and patient served per day were taken into consideration as candidates for the multivariable logistic regression analysis.

According to the multivariable logistic regression analysis results, respondents with master’s degrees were 2.13 times more likely to have good digital literacy than those with degrees and below educational levels (AOR = 2.13, 95% CI: 1.18–3.85). Health professionals with access to digital technology were 1.89 times more likely to have adequate digital literacy than those without (AOR = 1.89, 95% CI: 1.12–3.17). The current study also found that healthcare professionals who had received training in digital technology were 1.65 times more likely to have adequate digital literacy than their counterparts (AOR = 1.65, 95% CI: 1.05–2.59). Additionally, Health professionals with a favourable attitude toward digital health technology were 1.64 times more likely to have adequate digital literacy than those with unfavourable attitudes (AOR = 1.64, 95% CI: 1.02–2.68) Table 5.

**Table 5:** Factors associated with health professionals’ digital literacy level.

Variables		Digital literacy		COR (95% CI)	AOR (95% CI)
		Good (%)	Poor (%)		
Sex	Male	134 (32.6)	106 (25.8)	1.47 (0.99–2.18)	1.27 (0.82–1.96)
	Female	79 (19.2)	92 (22.4)	1	1
Age	30.3 ± 4.89			1.01 (0.97–1.05)	1.02 (0.97–1.08)
Education level	Master’s Degree and above	53 (12.9)	21 (5.1)	2.79 (1.61–4.83)	2.13 (1.18–3.85)*
	Degree and Below	160 (38.9)	177 (43.1)	1	1
Working experience	6.78 + 6.5			0.97 (0.94–1.01)	0.95 (0.91–1.01)
Access to digital technology	Yes	174 (42.3)	124 (30.2)	2.66 (1.69–4.18)	1.89 (1.12–3.17)*
	No	39 (9.5)	74 (18.0)	1	1
Internet access	Yes	184 (44.8)	148 (36.0)	2.14 (1.29–3.55)	1.28 (0.70–2.33)
	No	29 (7.1)	50 (12.2)	1	1
Training on digital technology	Yes	129 (31.4)	86 (20.9)	2.00 (1.35–2.96)	1.65 (1.05–2.59)*
	No	84 (20.4)	112 (27.3)	1	1
Staffs motivation	Yes	131 (31.9)	100 (24.3)	1.56 (1.05–2.31)	1.04 (0.66–1.64)
	No	82 (20.0)	98 (23.8)	1	1
Patients served per day	19.60 ± 10.4			1.01 (1.00–1.02)	1.01 (0.99–1.02)
Attitude	Good	169 (41.1)	138 (33.6)	1.67 (1.06–2.61)	1.64 (1.02–2.68)*
	Poor	44 (10.7)	60 (14.6)	1	1

* Significant at value of *p* < 0.05.

## Discussion

The results of this study revealed that 51.8% of health professionals have adequate digital literacy levels. This finding is consistent with studies in Ethiopia (5) and other studies conducted in Indonesia (7). However, our finding is lower compared to those of research studies which are conducted in Australia (33), Vietnam (34), and India (6). The discrepancy could result from variations in internet usage and ICT facilities among countries compared to Ethiopia. Evidence suggests that Ethiopia lags behind Africa’s average internet penetration rate (39%) (35).

The other explanation for this discrepancy could be that only roughly two-thirds (72%) of the study’s participants had access to digital technology, which is much fewer than in other wealthy nations like Australia (33) and may result in lower levels of digital literacy among health professionals in Ethiopia. In most affluent countries, digital health system implementation has been effective due to high computer literacy (33, 36). However, in underdeveloped countries, there is a low level of digital literacy among health workers, resulting in the stoppage of many e-health projects (37, 38). As a result, given the growing usage of technology in healthcare, it is critical that healthcare professionals are digitally literate (39, 40).

Respondents with master’s degrees were 2.13 times more likely to have adequate digital literacy than those with a degree and below educational levels. This finding is in line with previous research studies indicating that health professionals with higher education levels were more likely to have adequate digital literacy (5). This might be because health professionals with higher education levels were more likely to use digital technology for their education, such as research and data gathering tools, which were more frequently employed by master’s-level and more educated health professionals.

The current study also found that healthcare professionals receiving training in digital technology were 1.65 times more likely to have adequate digital literacy than their counterparts. The findings align with earlier studies conducted among nurses in Indonesia (7) and health professionals in Austral (33). This finding implies that general training on digital health technology and the specific eHealth software application that will be implemented may significantly impact how well the healthcare system adopts technology.

Health professionals with access to digital technology were 1.89 times more likely to be highly digitally literate than those without. This finding is in line with other research showing that medical personnel with access to digital devices, including computers, laptops, smartphones, and digital medical equipment, have a high level of digital literacy (41–44). This indicates that the Ethiopian ministry of health should increase the accessibility of digital health technology in the hospital setting to increase digital literacy among health professionals and successfully implement clinical health information systems.

Furthermore, this study found a significant association between the level of digital literacy and attitudes toward digital health technology. Health workers with a favourable attitude toward digital health technology were 1.64 times more likely to have good digital literacy than those with unfavourable attitudes. This conclusion is supported by earlier research showing that a positive attitude toward digital technology is associated with a greater likelihood of digital competence (33). This suggested that initiatives were required to alter health professionals’ attitudes toward digital health technology. This is because adopting a new mindset significantly contributes to efficiently integrating health information technologies into the healthcare system (45).

## Implication for policy and practice

This study has an implication for future digital health systems implementations. A possible way to raise the success rate of eHealth project implementations in Ethiopia is to increase computer accessibility, offer a training programme on digital health technology, and encourage a favourable attitude toward this technology. The study serves as a basis for the continuous implementation and customization of the electronic medical record at the University of Gondar Specialized and Comprehensive Hospital. Determining the level of digital literacy among the medical professionals working at the implementation location was the major objective to increase implementation success.

## Conclusion and recommendation

This study aimed to determine the digital literacy level of health professionals and the associated factors that have implications for future digital health systems implementation. The results of this study help us better understand the level of digital literacy among health professionals at the specialized hospital where an EMR system will be implemented. The results of this study demonstrated that health professionals have a relatively low level of digital literacy. It is suggested to increase computer accessibility, provide a training program on digital health technology, and promote a positive attitude toward this technology to improve the deployment of health information systems.

## Limitations and future works

The limitation of this study is that the data was collected through self-report, which could be prone to social desirability bias, so participants may have overestimated their responses. However, we tried to reduce this bias by making the questions as fair, neutral, and unthreatening as possible. Future work is required to determine the current status of adopting digital health tools by health professionals in Ethiopia. This will provide reliable data to assess interventions intended to increase the efficiency of various eHealth initiatives in Ethiopia.

## Data availability statement

The data presented in the study are included in the article/supplementary material, further inquiries can be directed to the corresponding author.

## Ethics statement

Ethical clearance and approval letters were obtained from the Institutional Review Board (IRB) of the University of Gondar with refernace number (Rfe. VP/RTT/05/571/2022). After explaining the study’s objective, each health professional signed a written consent form. The University of Gondar’s specialized hospital also obtained a letter of support. Confidentiality and privacy were ensured during data collection by keeping participants anonymous. The patients/participants provided their written informed consent to participate in this study.

## Author contributions

TY and MT made significant contributions to the conceptualization, design, data collection supervision, data analysis, interpretation, and writing of the publication. BT, AMa, HK, FN, EZ, AMe, GS, RG, HG, AS, and ST contributed equally to the data collection, analysis, interpretation, and manuscript review. All authors contributed to the article and approved the submitted version.

## Funding

The data collection for this study was funded by the University of Gondar (EMR customization and implementation project). The University of Gondar (EMR customization and implementation project) had no role in the study’s design, data collection, analysis, or interpretation of data, the decision to publish in peer-reviewed publications, or manuscript preparation.

## Conflict of interest

The authors declare that the research was conducted in the absence of any commercial or financial relationships that could be construed as a potential conflict of interest.

## Publisher’s note

All claims expressed in this article are solely those of the authors and do not necessarily represent those of their affiliated organizations, or those of the publisher, the editors and the reviewers. Any product that may be evaluated in this article, or claim that may be made by its manufacturer, is not guaranteed or endorsed by the publisher.

## Abbreviations

E-health: Electronic health
EMR: Electronic medical record
FMOH: Federal ministry of health
DHA: Digital health analysis
CBMP: Capacity building and mentorship project
DUP: Data use partnership
AOR: Adjusted odds ratio
COR: Crude odds ratio
CI: Confidence interval
SPSS: Statistical Package for Social Science
